# The Effects of Side-Chain Configurations of a Retro–Inverso-Type Inhibitor on the Human T-Cell Leukemia Virus (HTLV)-1 Protease

**DOI:** 10.3390/molecules27051646

**Published:** 2022-03-02

**Authors:** Chiyuki Awahara, Daiki Oku, Saki Furuta, Kazuya Kobayashi, Kenta Teruya, Kenichi Akaji, Yasunao Hattori

**Affiliations:** 1 Department of Medicinal Chemistry, Kyoto Pharmaceutical University, Yamashina-ku, Kyoto 607-8412, Japan; abelha417@gmail.com (C.A.); daikin12_brise@icloud.com (D.O.); saki.hr222@gmail.com (S.F.); kkoba@mb.kyoto-phu.ac.jp (K.K.); 2 Department of Neurochemistry, Tohoku University Graduate School of Medicine, Aoba-ku, Sendai 980-8575, Japan; kenta.teruya.d4@tohoku.ac.jp; 3 Center for Instrumental Analysis, Kyoto Pharmaceutical University, Yamashina-ku, Kyoto 607-8412, Japan

**Keywords:** HTLV-1 protease, inhibitor, retro–inverso conversion, hydroxyethylamine isostere

## Abstract

In this study, the effects of side-chain configurations of D-Ile residues of a retro–inverso (RI)-type inhibitor on the human T-cell leukemia virus type 1 (HTLV-1) protease containing a hydroxyethylamine dipeptide isostere were clarified. Prior to evaluation using the RI-type inhibitor, the effects of side-chain configurations of Ile residues of the substrate peptide on the HTLV-1 protease were examined to estimate the influence of side-chain configurations on enzyme activity. Based on the estimation of the influence of side-chain configurations on protease affinity, the RI-type inhibitors containing a D-allo-Ile residue in the corresponding substrate sequence, instead of a D-Ile residue, were synthesized via 9-fluorenylmethoxycarbonyl-based solid-phase peptide synthesis. Refolded recombinant HTLV-1 protease (1-116, L40I) was used for the simple and short evaluation of the inhibitory activities of the synthesized RI-type inhibitors. The results clearly indicated that mimicking the whole topology, comprising both the main- and side-chain structures of the parent inhibitor, is effective for the design of potent RI-modified protease inhibitors.

## 1. Introduction

Human T-cell leukemia virus type 1 (HTLV-1), the first reported human retrovirus, was isolated from adult patients with T-cell leukemia/lymphoma in 1980 [1]. It is estimated that 30 million people worldwide are infected with HTLV, and adult T-cell leukemia is prevalent in Japan [2]. Several treatments for adult T-cell leukemia, including antiviral therapy and combination chemotherapy, have been reported, but they show limited efficacy. The HTLV-1 encodes an aspartic protease, HTLV-1 protease, which processes its precursor polyprotein to yield a set of proteins, including the protease itself, which is necessary for viral replication [3]. Thus, HTLV-1 protease is a key protease in the replication of the virus; it is similar to the human immunodeficiency virus (HIV)-1 protease in acquired immune deficiency syndrome and is believed to be a suitable target for the development of inhibitors for therapeutic use, although a clinically effective inhibitor of HTLV-1 protease has not been developed. We previously reported the synthesis and structure–activity relationships of HTLV-1 protease inhibitors containing a transition state mimic, the hydroxyethylamine dipeptide isostere [4]. The results from that study showed that the configurations of the hydroxyl group mimicking the transition state and at the side-chain in the scissile site are essential for high potency [5,6] and that the amino acid residues at the P3–P1 sites, corresponding to the substrate sequence at the N terminal of the scissile position, form the core sequence for inhibitory activity. Based on these results, we introduced a D-amino acid, instead of the parent L-amino acid, into this core sequence to improve the in vivo stability of the substrate-based HTLV-1 protease inhibitor and demonstrated, for the first time, that the retro–inverso (RI) modification of an inhibitor containing a transition state mimic can retain inhibitory activity [7].

RI modification involves reversion of the amino acid sequence of the target peptide and replacement of each L-amino acid with the corresponding D-amino acid [8]. These double conversions are expected to retain a topography similar to that of the original peptide, which suggests that the RI-modified peptide may interact with its original target with an interaction mode similar to that of the parent peptide. Fully or partially RI-modified biologically active peptides retained the biological activity of their parent peptides [9,10,11]. Similar or improved interactions between RI-modified peptides and proteins or receptors have also been reported [12,13].

**Figure 1:**
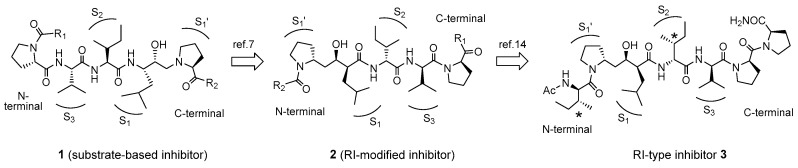
Retro–inverso (RI) modification of the human T-cell leukemia virus type 1 (HTLV-1) protease inhibitors containing a hydroxyethylamine isostere.

However, few applications of RI modification of protease inhibitors containing transition state mimics have been reported because of the difficulties in estimating the necessary stereo-structure at the scissile site. Simple conversion from L- to D-amino acids is not effective in protease inhibitors containing transition state mimic inhibitors, and a specific combination of stereo-structures at the scissile site is necessary for the design of an effective inhibitor. In our previous studies [7,14], we observed that a unique RI modification of an HTLV-1 protease inhibitor containing a specific hydroxyethylamine dipeptide isostere resulted in inhibitory activity, suggesting that this specific RI-modified inhibitor retained a topography similar to that of its parent inhibitor (Figure 1). Although the results strongly suggest that the combination of a specific stereo-structure at the scissile site with the RI conversion of the substrate sequence mimics the backbone topology of the inhibitor, the specific effect of the side-chain topology remains unknown. Thus, prior to evaluation using RI-type inhibitors, we examined whether the side-chain configurations at Ile residues with a β-site asymmetric carbon of the substrate affect the enzymatic activity of HTLV-1 protease. In this study, the proteolysis of several substrate-like peptides by HTLV-1 protease was examined, after which the effects of different topologies derived from the side-chain structure at the two Ile residues in the RI-type inhibitor were estimated. Additionally, to effectively evaluate the substrate affinity and inhibitory activity, refolded HTLV-1 protease produced by a recombinant procedure using *Escherichia coli* was employed in this study because the chemically synthesized protease [15] used in our previous studies needed to be folded in the individual assays, resulting in complicated procedures.

## 2. Results and Discussion

### 2.1. Preparation of Recombinant HTLV-1 Protease

HTLV-1 protease is an aspartic protease that consists of 125 amino acid residues and forms a homodimer in its active form, similar to the HIV-1 protease [16]. It processes its precursor protein to produce HTLV-1 protease and several other proteins necessary for the reproduction of viral particles [17]. Thus, autoproteolysis by the refolded protease consequently yields degraded fragments during the production of HTLV-1 protease by the recombinant procedure using *E. coli*. To prevent autoproteolysis, a mutation of Leu to Ile at position 40 was reported to be effective [18]. Moreover, there are practically no differences between the catalytic activities, such as in the k_cat_/K_m_ values, of native HTLV-1 (230.3 mM^−1^s^−1^) and mutated L40I (184.2 mM^−1^s^−1^) proteases. Additionally, a truncated HTLV-1 protease (positions 1-116) lacking nine C-terminal residues retains its protease activity, which is a characteristic property for the formation of a single crystal [19,20]. Considering these properties, in this study, a truncated mutant (L40I) HTLV-1 protease consisting of 116 amino acid residues was prepared using a conventional recombinant procedure in *E. coli*.

Recombinant HTLV-1 protease (1-116, L40I) in the inclusion bodies of *E. coli* was extracted using 6 M urea and refolded by dialysis against a buffer containing 50 mM acetate (pH 5.0), 1 mM DTT, and 1 mM EDTA. As the refolded recombinant protease (1-116, L40I) showed remarkable enzymatic activity for use in the evaluation of inhibitor potency, enzymatic activity was characterized by K_m_ and k_cat_ values obtained from the Hanes–Woolf plot (plotting (S)/v versus (S)) (Figure S1). The K_m_ value (32 ± 2 μM) of the recombinant protease (1-116, L40I) was 1.5 times higher than that (47 ± 5 μM) of the chemically synthesized HTLV-1 protease (1-125, L40I, C90A, C109A) used in our previous study [15], indicating that the recombinant protease had a higher affinity for the substrate. In contrast, the k_cat_ value (4.5 ± 0.3) remained at 55% of the corresponding k_cat_ value (8.2 ± 0.9) of the chemically synthesized protease, which shows that the turnover of the recombinant protease is lower than that of the synthetic protease. Although the catalytic efficiency (k_cat_/K_m_) of the recombinant protease was approximately 80% of that of the chemically synthesized protease, the folded recombinant protease showed sufficient potency for use in the subsequent evaluation of synthetic substrates and inhibitors without the time-consuming, individual refolding procedure required in previous experiments.

### 2.2. Synthesis and Evaluation of Allo-Ile-Containing HTLV-1 Protease Substrate

**Figure 2:**
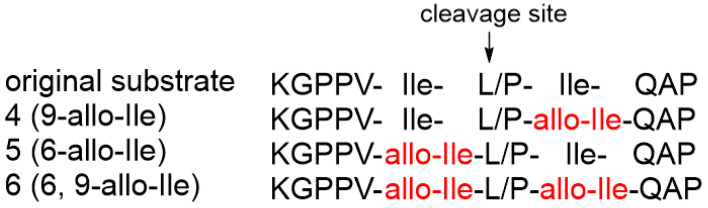
Sequences of HTLV-1 protease substrates containing the allo-Ile residues.

As the basis for the evaluation of the RI-type inhibitors, the effects of side-chain configurations of Ile residues of the HTLV-1 protease were first examined using the above recombinant protease. To evaluate the affinity of the HTLV-1 protease for substrates containing different side-chain configurations, three substrate peptides containing allo-Ile residue were synthesized by conventional Fmoc-based solid-phase peptide synthesis and TFA deprotection (Figure 2). The homogeneity of each synthesized substrate was confirmed using analytical HPLC (Figure S2) and LRESI–MS.

**Figure 3:**
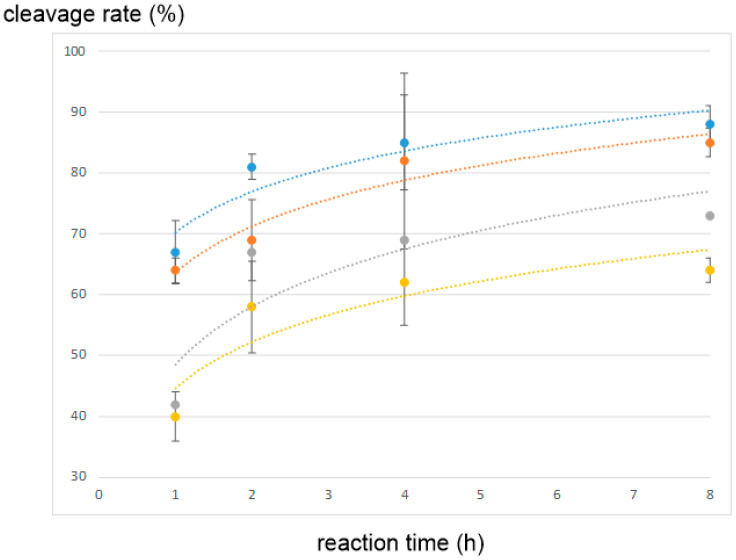
Cleavage reaction of the substrates containing allo-Ile residues by the HTLV-1 protease: blue, original substrate; orange, substrate **4**; gray, substrate **5**; yellow, substrate **6**.

Each synthesized substrate (4–6) was then treated with the recombinant HTLV-1 protease, and the decrease in each substrate level was monitored using analytical HPLC [15]. As summarized in Figure 3, substrate **4** containing an allo-Ile residue at the prime site was cleaved at a slightly lower rate than the original substrate, whereas substrate **5** containing an allo-Ile residue at the non-prime site and substrate **6** containing two allo-Ile residues at both sites clearly showed a lower cleavage rate than the original substrate, even after prolonged 8 h digestion (Figure S3; 83% for substrate **5** and 73% for substrate **6**). In addition, the time course of the cleavage reaction indicated that the replacement of an Ile residue at the non-prime site significantly reduced the protease efficiency. These results strongly suggest that the side-chain configurations of the Ile residue, especially at the non-prime site of the protease substrate, are accurately recognized by the HTLV-1 protease, and the entire topology of the substrate, including the side-chain, affects the affinity of the substrate for HTLV-1 protease.

### 2.3. Synthesis and Evaluation of the RI-Type HTLV-1 Protease Inhibitors

**Figure 4:**
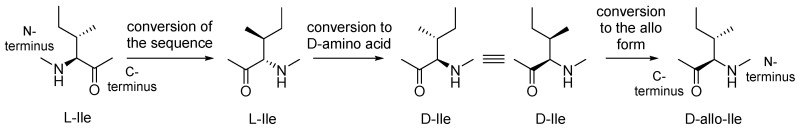
Optimization of the side-chain configurations of D-Ile residues of the RI-type inhibitors.

Based on the above results, the introduction of RI modification to the side-chain configuration was examined. The results suggested that simple conversion of L-Ile to D-Ile residues does not optimize the configuration at the side-chain of D-Ile in RI-type inhibitors, and an additional conversion of D-Ile to D-allo-Ile is suitable for topology holding, as shown in Figure 4.

**Scheme 1:**
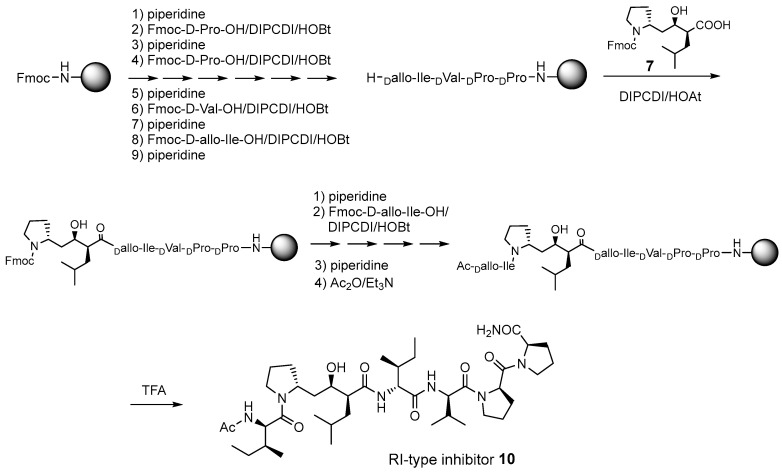
Synthetic scheme for the retro–inverso (RI)-type inhibitor **10**.

The synthetic scheme for the RI-type HTLV-1 protease inhibitors is shown in Scheme 1. A manual Fmoc-based solid-phase procedure was used to construct the peptide backbone. To incorporate the key transition-state mimic structure, a dipeptide-mimicking amino acid (**7**), with effective substituent configurations for the RI-modified inhibitor [14], was used. As the target RI-type inhibitor contains two D-Ile residues, four inhibitors were synthesized in combination with D-allo-Ile; **3** (1-D-Ile, 3-D-Ile), **8** (1-D-allo-Ile, 3-D-Ile), **9** (1-D-Ile, 3-D-allo-Ile), and **10** (1-D-allo-Ile, 3-D-allo-Ile).

Starting from the Rink amide resin [21], each D-amino acid derivative corresponding to the P2-P5 sites was successively introduced by the combination of 20% piperidine/DMF treatment and DIPCDI/HOBt-mediated coupling. For the condensation of a dipeptide isostere **7**, DIPCDI/HOAt-mediated coupling [21] was used. After condensation was complete, the terminal amino group of the resin was acetylated. Each peptide resin was treated with TFA to cleave each inhibitor from the resin. The crude product was purified using reverse-phase preparative HPLC to yield the desired derivatives **3** and **8**–**10** (overall yield, 11–19%). The homogeneity of each synthesized compound was confirmed using analytical HPLC (Figure S4), HRESI–MS, and ^1^H-NMR spectroscopy (Figure S5).

The inhibitory activities of the RI-type inhibitors obtained were examined using the recombinant HTLV-1 protease (1-116, L40I) and a substrate dodecapeptide, H-Lys-Gly Pro-Pro-Val-Ile-Leu-Pro-Ile-Gln-Ala-Pro-NH_2_, used in our previous procedure [14]. As the recombinant protease is refolded before use, mixing the protease with the substrate and inhibitor is sufficient for inhibitor evaluation; thus, the procedure is simple and short. The reaction mixture was directly analyzed by analytical HPLC with a C18 reversed-phase column. Each IC_50_ value of the RI-type inhibitor was obtained from the sigmoidal dose–response curve (Figure S6) calculated from the decrease in substrate concentration [14]. Although the observed sigmoidal curve indicated moderate IC_50_ values (80–100 μM), differences in the IC_50_ values of the RI-type inhibitors were confirmed. 

The IC_50_ value of RI-inhibitor **3** was 240 μM, which was approximately half of that (110 μM) estimated using the chemically synthesized protease (Table 1). This discrepancy is likely due to differences in the proteases used in this study. A relative excess of the recombinant protease, compared with the chemically synthesized protease, was used in a previous study, as the catalytic efficiency of the recombinant protease (1-116, L40I) was lower than that of the chemically synthesized protease. Using RI-inhibitor **3** as a reference, the effects of D-allo-Ile replacement were evaluated. Replacement of one of the two D-Ile residues (RI-inhibitor **8** (IC_50_ = 110 μM) and RI-inhibitor **9** (IC_50_ = 130 μM)) doubled the inhibitory activity, while the replacement of both D-Ile residues with D-allo-Ile (RI-inhibitor **10** (IC_50_ = 85 μM)) residues tripled the inhibitory activity. These results correspond well with the protease affinities of allo-Ile-containing substrates described above. Overall, these results indicate for the first time that the whole topology, comprising both the main- and side-chain structures, affects the protease affinity even in the RI-type conversion as in the original substrate.

**Figure 5:**
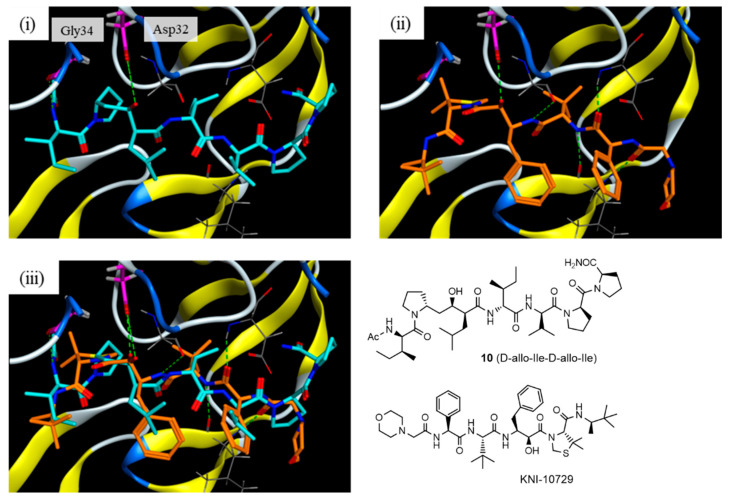
Docking model of compound **10** with the HTLV-1 protease: (**i**) interaction model for compound **10** (blue) with a Generalized-Born Volume Integral/Weighted Surface Area (GBVI/WSA) dG score of −11.0069 kcal/mol; (**ii**) X-ray crystal structure of KNI-10729 (orange); and (**iii**) overlapped model of compound **10** and KNI-10729.

The interactions of compound **10** with the HTLV-1 protease were further estimated using a docking model constructed using the Molecular Operating Environment (MOE) 2022.02 software package (Chemical Computing Group Inc., Montreal, QC, Canada). Template-guided docking by MOE using the norstatine-type inhibitor KNI-10729 (79% inhibition at 50 nM) [20] as a template (PDB 3LIX) estimated the interaction model, as shown in Figure 5. In the interaction model, the key hydroxyl group of **10** interacted with Asp32 at the active center of HTLV-1 protease as in KNI-10729, and the N-terminal acetamide group of P2′-D-allo-Ile interacted with the Gly34 of HTLV-1 protease. The topology of the side-chain structures of **10**, especially around the key hydroxyl group, roughly overlapped with that of the template inhibitor KNI-10729: P1-D-Leu to Phe, P2-D-allo-Ile to α-*t*-butyl Gly, P3-D-Val to phenyl Gly, P1′-D-Pro and the N-terminal acetamide group to the thioproline ring, and P2′-D-allo-Ile and N-terminal acetyl group to the C-terminal *t*-butyl group. These results suggest that the conversion of the side-chain configuration combined with the conversion of the main-chain configuration makes the entire topology of the RI-type inhibitor similar to that of the original ligand and improves its inhibitory activity.

## 3. Experimental

### 3.1. General

The reagents purchased were of commercial grade and used without further purification. Fmoc amino acid derivatives were purchased from Watanabe Chemical. Inc. (Hiroshima, Japan). The ^1^H-NMR spectra were recorded in CDCl_3_ using a Bruker Avance III 300 MHz spectrometer (Bruker Corporation, Billerica, MA, USA). Chemical shifts are expressed in ppm relative to tetramethylsilane (0.00 ppm). The coupling constants are given in Hz. Signal patterns are indicated as s, singlet; d, doublet; t, triplet; m, multiplet; and br, broadened peak. High-resolution (HR) and low-resolution (LR) mass spectra (MS) were recorded using a Shimadzu LCMS–IT–TOF instrument (ESI) (Shimadzu Corporation, Kyoto, Japan) with direct infusion.

Preparative HPLC was performed using a COSMOSIL 5C18-ARII (20 × 250 mm) (Nacalai Tesque, Inc., Kyoto, Japan) or YMC-TriartC18 (10 × 250 mm) (YMC. Co., Ltd., Kyoto, Japan) column, with a linear gradient of CH_3_CN containing 0.1% TFA in 0.1% aqueous TFA at a flow rate of 9.0 or 2.35 mL/min on a Hitachi ELITE LaChrom (Hitachi, Ltd., Tokyo, Japan) or Shimadzu Prominence system (OD, 220 or 210 nm) (Shimadzu Corporation, Kyoto, Japan). For analytical HPLC, a COSMOSIL 5C18-ARII (4.6 × 150 mm) or YMC-TriartC18 (4.6 × 250 mm) column was employed with a linear gradient of CH_3_CN containing 0.1% TFA in 0.1% aqueous TFA at a flow rate of 1.0 or 0.5 mL/min using a HITACHI ELITE LaChrom or Shimadzu Prominence system (OD, 220 or 210 nm).

### 3.2. Preparation of HTLV-1 Protease (1-116, L40I)

A plasmid encoding the HTLV-1 protease (1-125, L40I) with additional NdeI and BamHI restriction sites in the region corresponding to the N- and C-termini of the protease was constructed (pMA-HTLV-1 protease; GeneArt, Invitrogen, Waltham, MA, USA). Using the M13 primer set, a DNA fragment containing the HTLV-1 protease (1-125, L40I) was amplified. This fragment was digested with NdeI and BamHI and ligated into pTXB1 (New England Biolabs Inc., Ipswich, MA, USA) digested with the same restriction enzymes. DNA fragments for the HTLV-1 protease (1-116, L40I) with additional NdeI and BamHI restriction sites were amplified using the T7 primer and another primer, GGATCC-TATTAAGGGAGGTACAGGACGCC as forward and reverse primers, respectively. The DNA fragment encoding the HTLV-1 protease (1-116, L40I) was digested with NdeI and BamHI and ligated into pTXB1 (New England Biolabs Inc.) to obtain the plasmid vector p(HTLV-1 protease (1-116, L40I)).

HTLV-1 protease (1-116, L40I) was expressed in *E. coli* BL21(DE3) cells. The cell pellets were resuspended in a lysis buffer (20 mM Tris-HCl, and 300 mM NaCl, pH 7.5) containing phenylmethylsulfonyl fluoride. Cells were lysed by sonication and centrifuged. The pellet was resuspended in an equal volume of a buffer containing 6 M urea, 10 mM Tris, 5 mM EDTA, and 10 mM 2-mercaptoethanol. The resulting suspension was centrifuged again to remove the insoluble material. The supernatant was passed through a Hitrap Q FF column (5 mL). The flow-through was collected and dialyzed against 20 mM formic acid aqueous solution (MWCO 3000) overnight and then dialyzed again against a buffer containing 50 mM acetate (pH 5.0), 1 mM DTT, and 1 mM EDTA. Protein concentration was determined using the protein assay CBB kit (Nacalai Tesque, Inc., Kyoto, Japan), with bovine serum albumin solutions as reference solutions. The dialyzed and folded HTLV-1 protease (1-116, L40I) solutions were diluted to a final protein concentration of 35 µg/mL. The HTLV-1 protease (1-116, L40I) solution was stored at −20 °C.

### 3.3. Syntheses of Substrate Peptides

Three substrate-derived peptides were prepared by standard Fmoc-based solid-phase peptide synthesis (Fmoc-SPPS) using an automatic peptide synthesizer (PSSM-8; Shimadzu, Kyoto, Japan). For side-chain protection, the Boc group was used for Lys and the Trt group was used for Gln. The Fmoc-protected amino acids (3 equiv.) were coupled using HBTU (3 equiv.), HOBt·H_2_O (3 equiv.), and DIEA (6 equiv.) in DMF for 90 min. Fmoc deprotection was performed twice using 20% piperidine in DMF for 5 min.

Substrate-derived peptides (**4**–**6**) were cleaved from the corresponding resins by TFA treatment for 2 h at 25 °C and purified by preparative HPLC using a YMC-TriartC18 (10 × 250 mm) column with a linear gradient of CH_3_CN containing 0.1% TFA in 0.1% aqueous TFA at a flow rate of 2.35 mL/min with detection at 210 nm (CH_3_CN containing 0.1% TFA, 25–30%, 25 min for substrate **4** (9-allo-Ile); CH_3_CN containing 0.1% TFA, 30%, 15 min for substrate **5** (6-allo-Ile); CH_3_CN containing 0.1% TFA, 30–36%, 15 min for substrate **6** (6, 9-allo-Ile)).

**4** (9-allo-Ile), purity (HPLC area%) 96.4%, CH_3_CN containing 0.1% TFA, 10–90%, 30 min, 210 nm, LRESI MS, *m*/*z* 615 for [M+2H]^2+^ (615 calcd for C_59_H_103_N_15_O_13_).

**5** (6-allo-Ile), purity (HPLC area%) 97.8%, CH_3_CN containing 0.1% TFA, 10–90%, 30 min, 210 nm, LRESI MS, *m*/*z* 615 for [M+2H]^2+^ (615 calcd for C_59_H_103_N_15_O_13_).

**6** (6,9-allo-Ile), purity (HPLC area%) 95.7%, CH_3_CN containing 0.1% TFA, 10–90%, 30 min, 210 nm, LRESI MS, *m*/*z* 615 for [M+2H]^2+^ (615 calcd for C_59_H_103_N_15_O_13_).

### 3.4. Synthesis of RI-Peptide ***10***

Compound **10** was constructed by Fmoc-based solid-phase peptide synthesis using Fmoc-NH-SAL-resin [21] (0.47 mmol/g, 266 mg, 0.125 mmol) at 25 °C. Piperidine (20%) in DMF was added to the resin. After agitation for 20 min, the resin was washed with DMF. Fmoc-D-Pro-OH (63 mg, 0.188 mmol), HOBt·H_2_O (29 mg, 0.188 mmol), DIPCDI (29 µL, 0.188 mmol), and DIEA (33 µL, 0.188 mmol) in DMF were added, and the mixture was agitated for 2 h. The resin was filtered, washed with DMF and MeOH, and dried in vacuo to yield Fmoc-D-Pro-NH-SAL-resin, with a substitution rate of 0.089 mmol. After agitating with piperidine (20%) in DMF for 20 min, the resin was washed with DMF. Fmoc-D-Pro-OH (0.222 mmol), HOBt·H_2_O (34 mg, 0.222 mmol), DIPCDI (34 µL, 0.222 mmol), and DIEA (40 µL, 0.222 mmol) in DMF were added to this resin. The mixture was agitated for 2 h and washed with DMF. Completion of the coupling reaction was confirmed using the Kaiser ninhydrin test. Following piperidine treatment, coupling of Fmoc-D-Val-OH and Fmoc-D-allo-Ile-OH was carried out as described above. Next, coupling of **7** (60 mg, 0.134 mmol) was carried out with HOAt [22] (24 mg, 0.178 mmol), DIPCDI (27 µL, 0.178 mmol), and DIEA (32 µL, 0.178 mmol). The mixture was agitated for 18 h, and the resin was washed with DMF. The resulting resin was agitated with piperidine (20%) in DMF for 20 min. Fmoc-D-allo-Ile-OH (79 mg, 0.222 mmol) was coupled with HOBt·H_2_O (34 mg, 0.222 mmol), DIPCDI (34 µL, 0.222 mmol), and DIEA (40 µL, 0.222 mmol). The mixture was agitated for 2 h, and the resin was washed with DMF. The resulting resin was agitated with piperidine (20%) in DMF for 20 min. After agitating with Ac_2_O (67 µL, 0.712 mmol) and Et_3_N (0.10 mL, 0.712 mmol) in DMF for 10 min, the product resin was washed with DMF and MeOH. TFA was added to the dried resin and the mixture was agitated for 2 h at 25 °C. The mixture was filtered, and TFA was removed under reduced pressure. Ether was added, and the precipitate was purified via preparative HPLC (CH_3_CN containing 0.1% TFA, 30–50%, 40 min, 220 nm) to yield **10** (8.8 mg, yield 19% from Fmoc-D-Pro-NH-SAL-resin, purity (HPLC area%) > 99.9%, CH_3_CN containing 0.1% TFA, 30–50%, 40 min, 220 nm) as a white powder; ^1^H NMR: δ 0.82-1.06 (m, 24H), 1.09–1.42 (m, 6H), 1.45–1.85 (m, 8H), 1.88–2.06 (m, 6H), 1.97 (s, 1.8H), 2.01 (s, 1.2H), 2.08–2.28 (m, 4H), 2.33–2.60 (m, 2H), 3.49–3.61 (m, 4H), 3.65–3.77 (m, 2H), 3.82–3.88 (m, 0.33H), 3.89–4.04 (m, 0.67H), 4.11–4.19 (m, 0.67H), 4.26–4.29 (m, 0.33H), 4.33–4.47 (m, 2H), 4.55–4.69 (m, 3H), 5.30 (m, 0.67H), 6.56 (m, 0.33H), 6.62–6.69 (m, 1H), 6.75–6.78 (m, 1H), 6.86 (br d, J = 9.7 Hz, 0.67H), 7.19 (br d, J = 9.3 Hz, 0.33H), 7.74 (br d, J = 9.1 Hz, 0.67H), 7.98 (m, 0.33H); HRESI MS, m/z 812.5261 for [M+Na]^+^ (812.5256 calcd for C_41_H_71_N_7_NaO_8_).

### 3.5. Synthesis of ***3***, ***8***, and ***9***

These compounds were similarly synthesized according to the above procedure.

**RI-Peptide 3**

White powder; yield 16% (11 mg), purity (HPLC area%) >99.9%, CH_3_CN containing 0.1% TFA, 40–60%, 40 min, 220 nm: ^1^H NMR: δ 0.85–0.96 (m, 21H), 1.03 (d, J = 6.7 Hz, 1.65H), 1.05 (d, J = 6.7 Hz, 1.35H), 1.09–1.17 (m, 2H), 1.22–1.64 (m, 6H), 1.68–1.94 (m, 9H), 1.97 (s, 1.65H), 2.00 (s, 1.35H), 1.97–2.07 (m, 3H), 2.09–2.28 (m, 4H), 2.36–2.55 (m, 2H), 3.47–3.76 (m, 5H), 3.93–4.29 (m, 3H), 4.34–4.52 (m, 3H), 4.55–4.65 (m, 2H), 5.36 (m, 0.55H), 6.59–6.62 (m, 1H), 6.71–6.74 (m, 1H), 6.80 (m, 0.45H), 6.87 (br d, J = 9.7 Hz, 0.55H), 7.14–7.17 (m, 0.45H), 7.61–7.74 (m, 0.55H), 8.04 (m, 0.45H); HRESI MS, *m*/*z* 812.5251 for [M+Na]^+^ (812.5256 calcd for C_41_H_71_N_7_NaO_8_).

**RI-Peptide 8**

White powder; yield 17% (8.3 mg), purity (HPLC area%) >99.9%, CH_3_CN containing 0.1% TFA, 30–50%, 40 min, 220 nm: ^1^H NMR: δ 0.83–0.96 (m, 18H), 0.97 (t, J = 6.3 Hz, 3H), 1.04 (t, J = 6.3 Hz, 3H), 1.10–1.16 (m, 2H), 1.26–1.39 (m, 4H), 1.41–1.86 (m, 8H), 1.87–2.07 (m, 6H), 1.97 (s, 1.8H), 2.01 (s, 1.2H), 2.09–2.28 (m, 3H), 2.30–2.62 (m, 3H), 3.52–3.58 (m, 3H), 3.63–3.76 (m, 2H), 3.82–4.29 (m, 3H), 4.32–4.49 (m, 3H), 4.52–4.69 (m, 2H), 5.26 (m, 0.67H), 6.37–6.39 (m, 0.33H), 6.60 (br d, J = 8.4 Hz, 0.67H), 6.69–6.75 (m, 1.33H), 6.84 (br d, J = 9.6 Hz, 0.67H), 7.02 (br d, J = 9.1 Hz, 0.33H), 7.54 (br d, J = 9.3 Hz, 0.67H), 8.01 (m, 0.33H); HRESI MS, *m*/*z* 812.5268 for [M+Na]^+^ (812.5256 calcd for C_41_H_71_N_7_NaO_8_).

**RI-Peptide 9**

White powder; yield 11% (5.0 mg), purity (HPLC area%) >99.9%, CH_3_CN containing 0.1% TFA, 30–50%, 40 min, 220 nm: ^1^H NMR: δ 0.82–0.95 (m, 19H), 0.99 (d, J = 6.7 Hz, 2H), 1.04 (d, J = 6.7 Hz, 2H), 1.05 (d, J = 6.7 Hz, 1H), 1.08–1.35 (m, 5H), 1.36–1.85 (m, 9H), 1.88–2.04 (m, 6H), 1.96 (s, 1.8H), 1.99 (s, 1.2H), 2.09–2.31 (m, 4H), 2.35–2.60 (m, 2H), 3.46–3.64 (m, 4H), 3.67–3.77 (m, 2H), 3.92–4.29 (m, 2H), 4.43–4.48 (m, 2H), 4.51–4.65 (m, 3H), 5.25 (m, 0.67H), 6.62 (br d, J = 8.3 Hz, 0.67H), 6.69 (br d, J = 9.6 Hz, 1H), 6.74–6.77 (m, 0.33H), 6.87–6.91 (m, 1H), 7.34 (m, 0.33H), 7.69 (br d, J = 9.8 Hz, 0.67H), 7.97–7.98 (m, 0.33H); HRESI MS, *m*/*z* 812.5260 for [M+Na]^+^ (812.5256 calcd for C_41_H_71_N_7_NaO_8_).

### 3.6. Measurement of Inhibitory Activity

A mixture of recombinant HTLV-1 (1-116, L40I) protease at an enzyme concentration of 70 nM, the substrate peptide H-Lys-Gly- Pro-Pro-Val-Ile-Leu-Pro-Ile-Gln-Ala-Pro-NH_2_ at a concentration of 120 μM, and the synthetic RI-type inhibitor (1 μL) at a concentration of 39~2500 μM was incubated in a phosphate buffer (pH 6.5, 20μL) at 37 °C for 2 h. The reaction mixture was analyzed on a Cosmosil 5C18-ARII column (4.6 × 150 mm), employing a linear gradient of CH_3_CN (10–40%, 30 min) in aqueous 0.1% TFA. Each IC_50_ value was obtained from a sigmoidal dose–response curve obtained from the decrease in the substrate in the reaction mixture (Figure S3). Each experiment was repeated three times.

### 3.7. Construction of Docking Models

The docking model was constructed using the X-ray crystal structure of a complex of HTLV-1 protease and the inhibitor, KNI-10729 (PDB 3LIX), as a template. The possible binding mode was obtained using an automated template-guided docking protocol with the Amber10:EHT force field in the MOE 2022.02 software package (Chemical Computing Group Inc., Montreal, QC, Canada).

## 4. Conclusions

Recombinant HTLV-1 protease (1-116, L40I) has nearly the same enzymatic properties as the chemically synthesized HTLV-1 protease employed in our previous studies, as well as the same effects on HTLV-1 protease inhibitors. The recombinant protease is refolded in the isolation process from the inclusion body formed in *E. coli* and is effective for the simple and short evaluation of protease inhibitors. It was confirmed, for the first time, that the configuration at the side chain of two Ile residues of the substrate sequence for the HTLV-1 protease affects the enzymatic potency. Conversion of Ile to allo-Ile at both positions of the substrate lowered the protease affinity most significantly. Moreover, the conversion of the configuration at the corresponding side chain of the two D-Ile residues in the RI-type inhibitor for HTLV-1 protease increased its inhibitory activity. RI-type inhibitors with both D-Ile residues converted to D-allo-Ile residues showed more efficient inhibitory activities than the RI-type inhibitors with only one converted D-Ile residue. These results, combined with docking simulation studies, suggest that the entire topology, including the main- and side-chain structures of the parent compound, mimics that of the RI-modified protease inhibitor. Therefore, these results regarding the effects of side-chain configurations after RI modification reveal a potential new avenue for the study of RI-type inhibitors.

## Figures and Tables

**Table 1:** IC_50_ values of the RI-type inhibitors.

Compound	IC_50_ (μM)
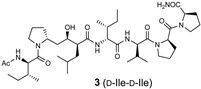	240
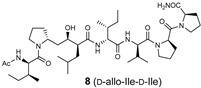	110
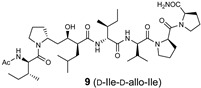	130
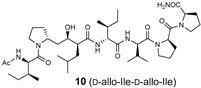	85

## Data Availability

All data described in this article has been included in the Supplementary Materials.

## Supplementary Materials

The following supporting information can be downloaded. Figure S1: Estimation of K_m_ and k_cat_ values of the recombinant HTLV-1 [1-116, L40I] protease; Figure S2: HPLC profiles of substrate derivatives; Figure S3: Cleavage rate of substrate derivatives; Figure S4: HPLC profiles of the synthetic RI-type inhibitors; Figure S5: The ESI mass spectra and NMR spectra of the synthetic RI-type inhibitors; Figure S6: Sigmoidal curve for each RI-type inhibitors.

## Sample Availability

Samples of the compounds have been used in this study and are not available from the authors.

## Abbreviation

DIPCDI, diisopropylcarbodiimide; DIEA, diisopropylethyl amine; DMF, dimethylformamide; DTT, dithiothreitol; EDTA, ethylenediaminetetraacetic acid; Fmoc, 9-fluorenylmethoxycarbonyl; HBTU, 2-(1H-Benzotriazole-1-yl)-1,1,3,3-tetramethyluronium hexafluorophosphate; HIV, human immunodeficiency virus; HOAt, 1-hydroxy-7-azabenzotriazole; HOBt, 1-hydroxybenzotriazole; HPLC, high-performance liquid chromatography; HRESI–MS, high-resolution electrospray ionization–mass spectrometry; HTLV-1, human T-cell leukemia virus type 1; LRESI–MS, low-resolution electrospray ionization–mass spectrometry; MOE, molecular operating environment; RI, retro–inverso; Rink amide resin, 4-(2,4-dimethoxyphenyl-Fmoc-aminomethyl)-phenoxy resin; TFA, trifluoroacetic acid.
