# Correction: Awahara et al. The Effects of Side-Chain Configurations of a Retro–Inverso-Type Inhibitor on the Human T-Cell Leukemia Virus (HTLV)-1 Protease. *Molecules* 2022, *27*, 1646

**DOI:** 10.3390/molecules30020334

**Published:** 2025-01-16

**Authors:** Chiyuki Awahara, Daiki Oku, Saki Furuta, Kazuya Kobayashi, Kenta Teruya, Kenichi Akaji, Yasunao Hattori

**Affiliations:** 1 Department of Medicinal Chemistry, Kyoto Pharmaceutical University, Yamashina-ku, Kyoto 607-8412, Japan; abelha417@gmail.com (C.A.); daikin12_brise@icloud.com (D.O.); saki.hr222@gmail.com (S.F.); kkoba@mb.kyoto-phu.ac.jp (K.K.); 2 Department of Neurochemistry, Tohoku University Graduate School of Medicine, Aoba-ku, Sendai 980-8575, Japan; kenta.teruya.d4@tohoku.ac.jp; 3 Center for Instrumental Analysis, Kyoto Pharmaceutical University, Yamashina-ku, Kyoto 607-8412, Japan

**Figure Legend**

In the original publication [1], there was a mistake in the legend for Figure 5. The dG score must be changed. The correct legend appears below. **Figure 5.** Docking model of compound **10** with the HTLV-1 protease: (i) interaction model for compound **10** (blue) with a Generalized-Born Volume Integral/Weighted Surface Area (GBVI/WSA) dG score of −11.0069 kcal/mol; (ii) X-ray crystal structure of KNI-10729 (orange); and (iii) overlapped model of compound 10 and KNI-10729.

**Error in Figures/Table**

In the original publication [1], there were five mistakes in Figure 1, Figure 4, Scheme 1, Table 1, and Figure 5 as published. The structure of the side-chain of L-Ile, D-Ile, and D-allo-Ile were inverted. The corrected Figure 1, Figure 4 and Figure 5, Scheme 1, and Table 1 appear below.

**Figure 1:**
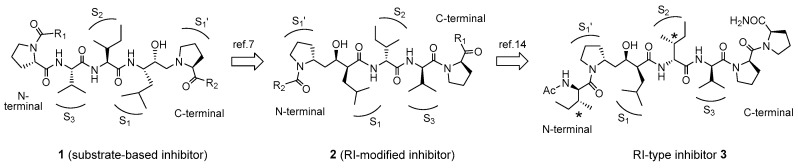
Retro–inverso (RI) modification of the human T-cell leukemia virus type 1 (HTLV-1) protease inhibitors containing a hydroxyethylamine isostere.

**Figure 4:**
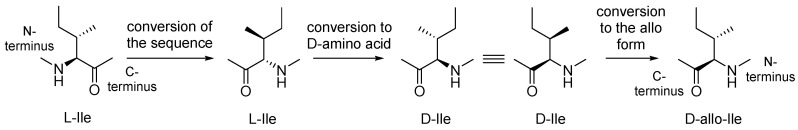
Optimization of the side-chain configurations of D-Ile residues of the RI-type inhibitors.

**Scheme 1:**
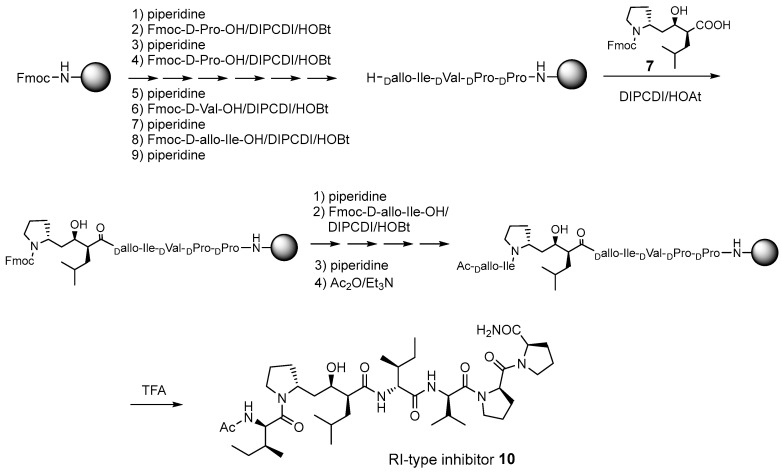
Synthetic scheme for the retro–inverso (RI)-type inhibitor **10**.

**Table 1:** IC_50_ values of the RI-type inhibitors.

Compound	IC_50_ (μM)
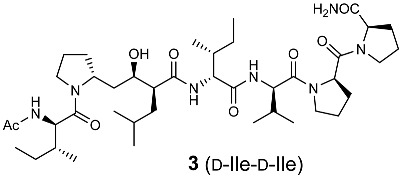	240
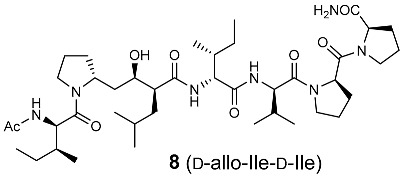	110
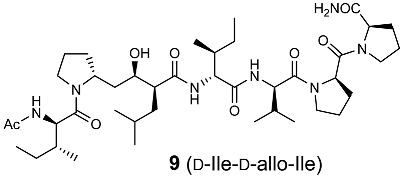	130
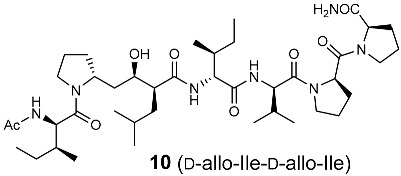	85

**Figure 5:**
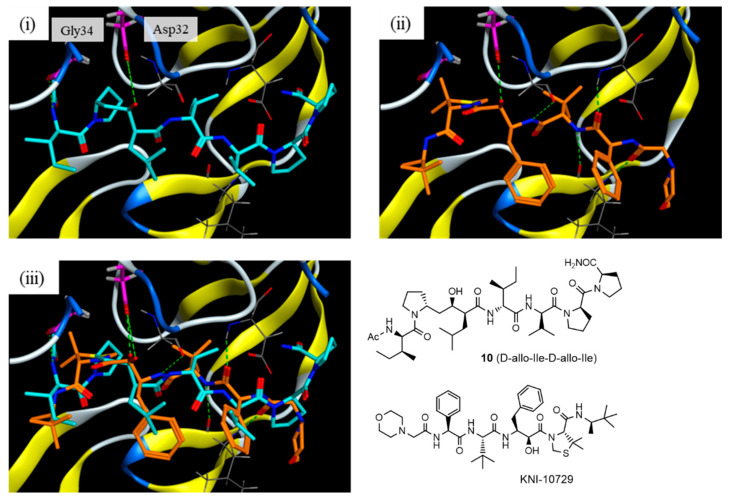
Docking model of compound **10** with the HTLV-1 protease: (**i**) interaction model for compound **10** (blue) with a Generalized-Born Volume Integral/Weighted Surface Area (GBVI/WSA) dG score of −11.0069 kcal/mol; (**ii**) X-ray crystal structure of KNI-10729 (orange); and (**iii**) overlapped model of compound **10** and KNI-10729.

## Text Correction

There were two errors in the original publication [1]. The structure of the side-chain of D-Ile and D-allo-Ile were inverted. Thus, a docking model needed to be reconstructed using the new version of Molecular Operating Environment (MOE).

Corrections were made to 2. Results and Discussion, 2.3. Synthesis and Evaluation of the RI-Type HTLV-1 Protease Inhibitors, Paragraph 6 and 3. Experimental, 3.7. Construction of Docking Models:

2. Results and Discussion, 2.3. Synthesis and Evaluation of the RI-Type HTLV-1 Protease Inhibitors, Paragraph 6

The interactions of compound **10** with the HTLV-1 protease were further estimated using a docking model constructed using the Molecular Operating Environment (MOE) 2022.02 software package (Chemical Computing Group Inc., Montreal, QC, Canada). Template-guided docking by MOE using the norstatine-type inhibitor KNI-10729 (79% inhibition at 50 nM) [20] as a template (PDB 3LIX) estimated the interaction model, as shown in Figure 5. In the interaction model, the key hydroxyl group of **10** interacted with Asp32 at the active center of HTLV-1 protease as in KNI-10729, and the N-terminal acetamide group of P2′-D-allo-Ile interacted with the Gly34 of HTLV-1 protease. The topology of the side-chain structures of **10**, especially around the key hydroxyl group, roughly overlapped with that of the template inhibitor KNI-10729: P1-D-Leu to Phe, P2-D-allo-Ile to α-*t*-butyl Gly, P3-D-Val to phenyl Gly, P1′-D-Pro and the N-terminal acetamide group to the thioproline ring, and P2′-D-allo-Ile and N-terminal acetyl group to the C-terminal *t*-butyl group. These results suggest that the conversion of the side-chain configuration combined with the conversion of the main-chain configuration makes the entire topology of the RI-type inhibitor similar to that of the original ligand and improves its inhibitory activity.

3. Experimental, 3.7. Construction of Docking Models

The docking model was constructed using the X-ray crystal structure of a complex of HTLV-1 protease and the inhibitor, KNI-10729 (PDB 3LIX), as a template. The possible binding mode was obtained using an automated template-guided docking protocol with the Amber10:EHT force field in the MOE 2022.02 software package (Chemical Computing Group Inc., Montreal, QC, Canada).

The authors state that the scientific conclusions are unaffected. This correction was approved by the Academic Editor. The original publication has also been updated.
