# Supplementary material for: The Effects of Side-Chain Configurations of a Retro–Inverso-Type Inhibitor on the Human T-Cell Leukemia Virus (HTLV)-1 Protease
Source: Molecules. 2022 Mar 2;27(5):1646. doi: 10.3390/molecules27051646 (PMC8911550; doi:10.3390/molecules27051646)

Supplementary Materials

The Effects of Side-Chain Configurations of a Retro–Inverso-Type Inhibitor on the Human T-Cell Leukemia Virus (HTLV)-1 Protease

Chiyuki Awahara [1], Daiki Oku [1], Saki Furuta [1], Kazuya Kobayashi [1], Kenta Teruya [2], Kenichi Akaji [1,*,†], and Yasunao Hattori [3,*,†]

[1] Department of Medicinal Chemistry, Kyoto Pharmaceutical University, Yamashina-ku, Kyoto 607-8412, Japan; abelha417@gmail.com (C.A.); daikin12_brise@icloud.com (D.O.); saki.hr222@gmail.com (S.F.); kkoba@mb.kyoto-phu.ac.jp (K.K.)
[2] Department of Neurochemistry, Tohoku University Graduate School of Medicine, Aoba-ku,
Sendai 980-8575, Japan; kenta.teruya.d4@tohoku.ac.jp
[3] Center for Instrumental Analysis, Kyoto Pharmaceutical University, Yamashina-ku, Kyoto 607-8412, Japan
* Correspondence: akaji@mb.kyoto-phu.ac.jp (K.A.); hattori@mb.kyoto-phu.ac.jp (Y.H.);
Tel./Fax: +81-75595-4604 (K.A.)
† These authors contributed equally to this work.

Figure S1. Estimation of K_m and k_{cat} values of the recombinant HTLV-1 [1-116, L40I] protease
Figure S2. HPLC profiles of substrate derivatives
Figure S3. Cleavage rate of substrate derivatives
Figure S4. HPLC profiles of the synthetic RI-type inhibitors
Figure S5. The ESI mass spectra and NMR spectra of the synthetic RI-type inhibitors
Figure S6. Sigmoidal curve for each RI-type inhibitors

*Corresponding author; Yasunao Hattori and Kenichi Akaji are equally contributed on this study
Kenichi Akaji, Ph.D.
Department of Medicinal Chemistry, Kyoto Pharmaceutical University, Kyoto 607-8412, Japan
Tel/Fax: +81-75-595-4604. E-mail: akaji@mb.kyoto-phu.ac.jp

Figure S1. Estimation of K_m and k_{cat} values of the recombinant HTLV-1 [1-116, L40I] protease

										[Sub]stock		[Enz]stock													
sub-dependency									No.	1628 µM		0.028 µM		area	absolute amount		Product			V		graph			incub.
No.	sub-I	water	enz	x2buf	total	dillut	smpl	auto sampl		[Sub]o/µM	[Enz]/µM	ab[Sub]/nmo	[area]init	Rea-Sub	Sub/nmol	Pro/nmol	%	conc/µM		µM/min.		S	S/V		min.
0	1.2	11.3	0	12.5	25	100	125	90	0	78.144	0	1.406592	889625	889625	1.41	0.00	0.00	0.00							
1	1.2	5.3	6	12.5	25	100	125	90	1	78.144	0.00672	1.406592	889625	707513	1.12	0.29	20.47	20.00		1.333		78	59		15
2	1.2	5.3	6	12.5	25	100	125	90	2	55.817	0.00672	1.004709	635446	476543	0.75	0.25	25.01	17.45		1.246		56	45		14
3	1.2	5.3	6	12.5	25	100	125	90	3	39.869	0.00672	0.717649	453890	335119	0.53	0.19	26.17	13.04		1.003		40	40		13
4	1.2	5.3	6	12.5	25	100	125	90	4	28.478	0.00672	0.512606	324207	246214	0.39	0.12	24.06	8.56		0.714		28	40		12
5	1.2	5.3	6	12.5	25	100	125	90	5	20.342	0.00672	0.366147	231577	149308	0.24	0.13	35.53	9.03		0.821		20	25		11
6	1.2	5.3	6	12.5	25	100	125	90	6	14.530	0.00672	0.261534	165412	108541	0.17	0.09	34.38	6.24		0.624		15	23		10
0	1.2	11.3	0	12.5	25	100	125	90	0	78.144	0	1.406592	921256	921256	1.41	0.00	0.00	0.00							
1	1.2	5.3	6	12.5	25	100	125	90	1	78.144	0.00672	1.406592	921256	734721	1.12	0.28	20.25	19.78		1.319		78	59		15
2	1.2	5.3	6	12.5	25	100	125	90	2	55.817	0.00672	1.004709	658040	503147	0.77	0.24	23.54	16.42		1.173		56	48		14
3	1.2	5.3	6	12.5	25	100	125	90	3	39.869	0.00672	0.717649	470029	331188	0.51	0.21	29.54	14.72		1.132		40	35		13
4	1.2	5.3	6	12.5	25	100	125	90	4	28.478	0.00672	0.512606	335735	239272	0.37	0.15	28.73	10.23		0.852		28	33		12
5	1.2	5.3	6	12.5	25	100	125	90	5	20.342	0.00672	0.366147	239810	174033	0.27	0.10	27.43	6.97		0.634		20	32		11
6	1.2	5.3	6	12.5	25	100	125	90	6	14.530	0.00672	0.261534	171293	107890	0.16	0.10	37.01	6.72		0.672		15	22		10
0	1.2	11.3	0	12.5	25	100	125	90	0	78.144	0	1.406592	932307	932307	1.42	-0.02	-1.20	-1.17							
1	1.2	5.3	6	12.5	25	100	125	90	1	78.144	0.00672	1.406592	921256	759011	1.16	0.25	17.61	17.20		1.147		78	68		15
2	1.2	5.3	6	12.5	25	100	125	90	2	55.817	0.00672	1.004709	658040	536626	0.82	0.19	18.45	12.87		0.920		56	61		14
3	1.2	5.3	6	12.5	25	100	125	90	3	39.869	0.00672	0.717649	470029	343584	0.52	0.19	26.90	13.41		1.031		40	39		13
4	1.2	5.3	6	12.5	25	100	125	90	4	28.478	0.00672	0.512606	335735	224000	0.34	0.17	33.28	11.85		0.987		28	29		12
5	1.2	5.3	6	12.5	25	100	125	90	5	20.342	0.00672	0.366147	239810	177460	0.27	0.10	26.00	6.61		0.601		20	34		11
6	1.2	5.3	6	12.5	25	100	125	90	6	14.530	0.00672	0.261534	171293	128792	0.20	0.06	24.81	4.51		0.451		15	32		10

run-1

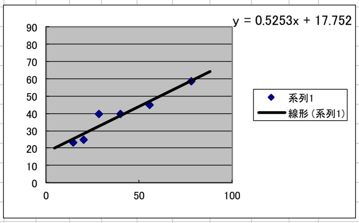

run-2

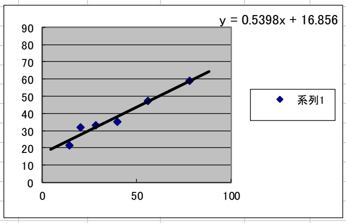

run-3

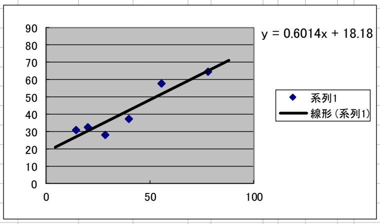

	a	b	Km(µM)	V(µM/min)	kcat/sec	kcat/Km
1	0.5253	17.752	33.8	1.90	4.72141	0.1397
2	0.5398	16.856	31.2	1.85	4.59459	0.1471
3	0.6014	18.18	30.2	1.66	4.12398	0.1364

	Km	kcat/sec
	33.8	4.72
	31.2	4.59
	30.2	4.12
average	31.7	4.48
SD	1.9	0.32

Figure S2. HPLC profiles of substrate derivatives

4 (9-allo-Ile)

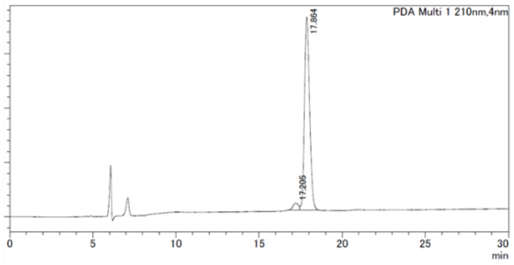

5 (6-allo-Ile)

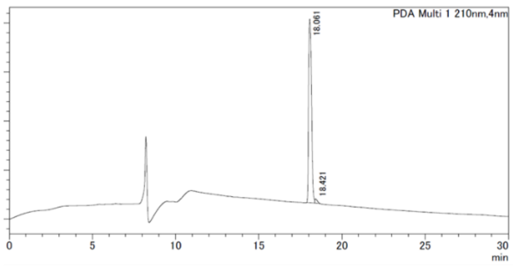

6 (6,9-allo-Ile)

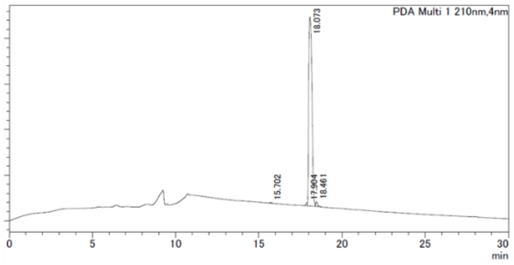

Figure S3. Cleavage rate of substrate derivatives

sub-0	KGPPV- Ile- L/P- Ile- QAP
sub-1	KGPPV- Ile- L/P-alloIle-QAP
sub-2	KGPPV-alloIle-L/P- Ile- QAP
sub-3	KGPPV-alloIle-L/P-alloIle-QAP

切断率（%）

	1	2	4	8	8 h
sub-0	67	81	85	88	100
sub-1	64	69	82	85	97
sub-2	42	67	69	73	83
sub-3	40	58	62	64	73

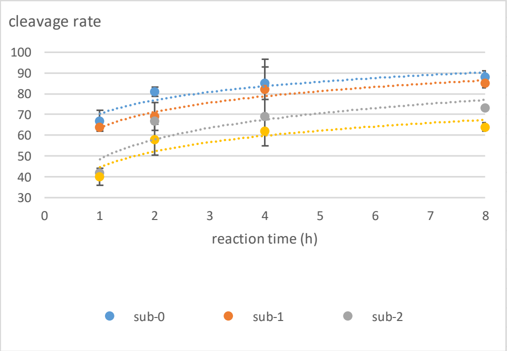

sub-0	run-1	run-2	run-3		ave	STD
1 hr	73	64	64	1	67.0	5.2
2	80	83	79	2	80.7	2.1
4	91	87	76	4	84.7	7.8
8	85	91	87	8	87.7	3.1

sub-1	run-1	run-2	run-3		ave	
1	65	66	62	1	64.3	2.1
2	72	61	73	2	68.7	6.7
4	91	89	65	4	81.7	14.5
8	88	84	84	8	85.3	2.3

sub-2	run-1	run-2	run-3		ave	
1	53	43	30	1	42.0	11.5
2	66	71	64	2	67.0	3.6
4	65	75	66	4	68.7	5.5
8	66	79	75	8	73.3	6.7

sub-3	run-1	run-2	run-3		ave	
1	44	40	36	1	40.0	4.0
2	58	65	50	2	57.7	7.5
4	55	69	62	4	62.0	7.0
8	62	64	66	8	64.0	2.0

Figure S4. HPLC profiles of the synthetic RI-type inhibitors

3 (D-Ile-D-Ile); IC_{50} = 240 μM

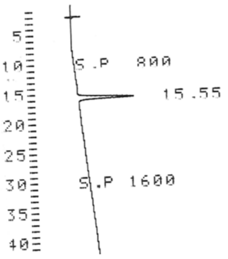

8 (D-allo-Ile-D-Ile); IC_{50} = 110 μM

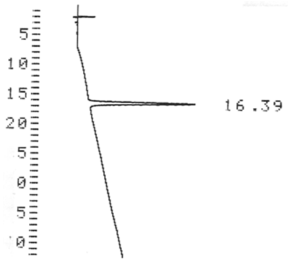

9 (D-Ile-D-allo-Ile); IC_{50} = 130 μM

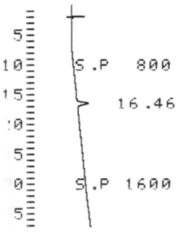

10 (D-allo-Ile-D-allo-Ile); IC_{50} = 85 μM

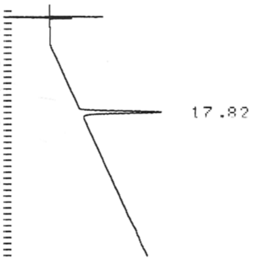

Figure S5. The ESI mass spectra and NMR spectra of the synthetic RI-type inhibitors

RI-peptide 10

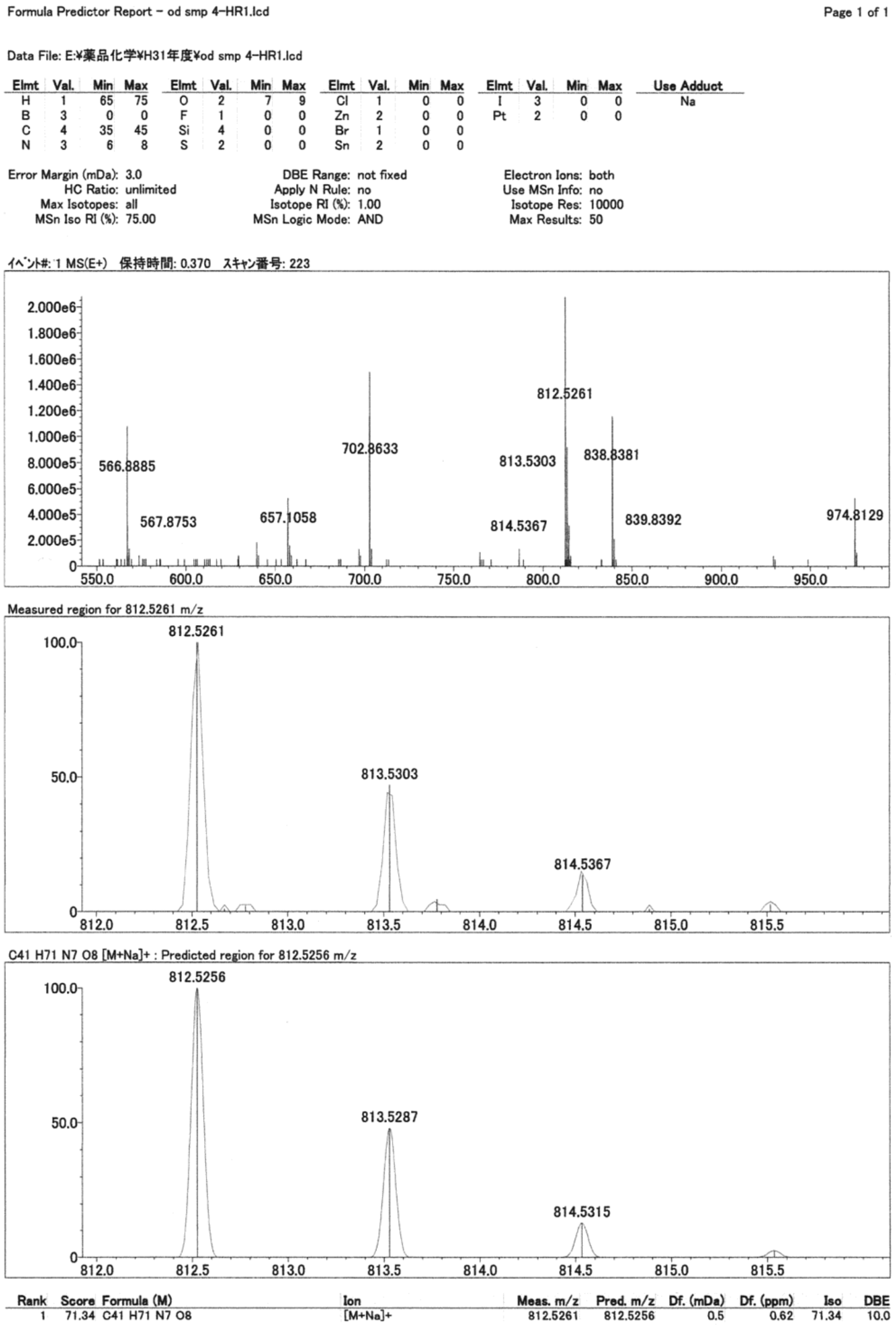

RI-peptide 10

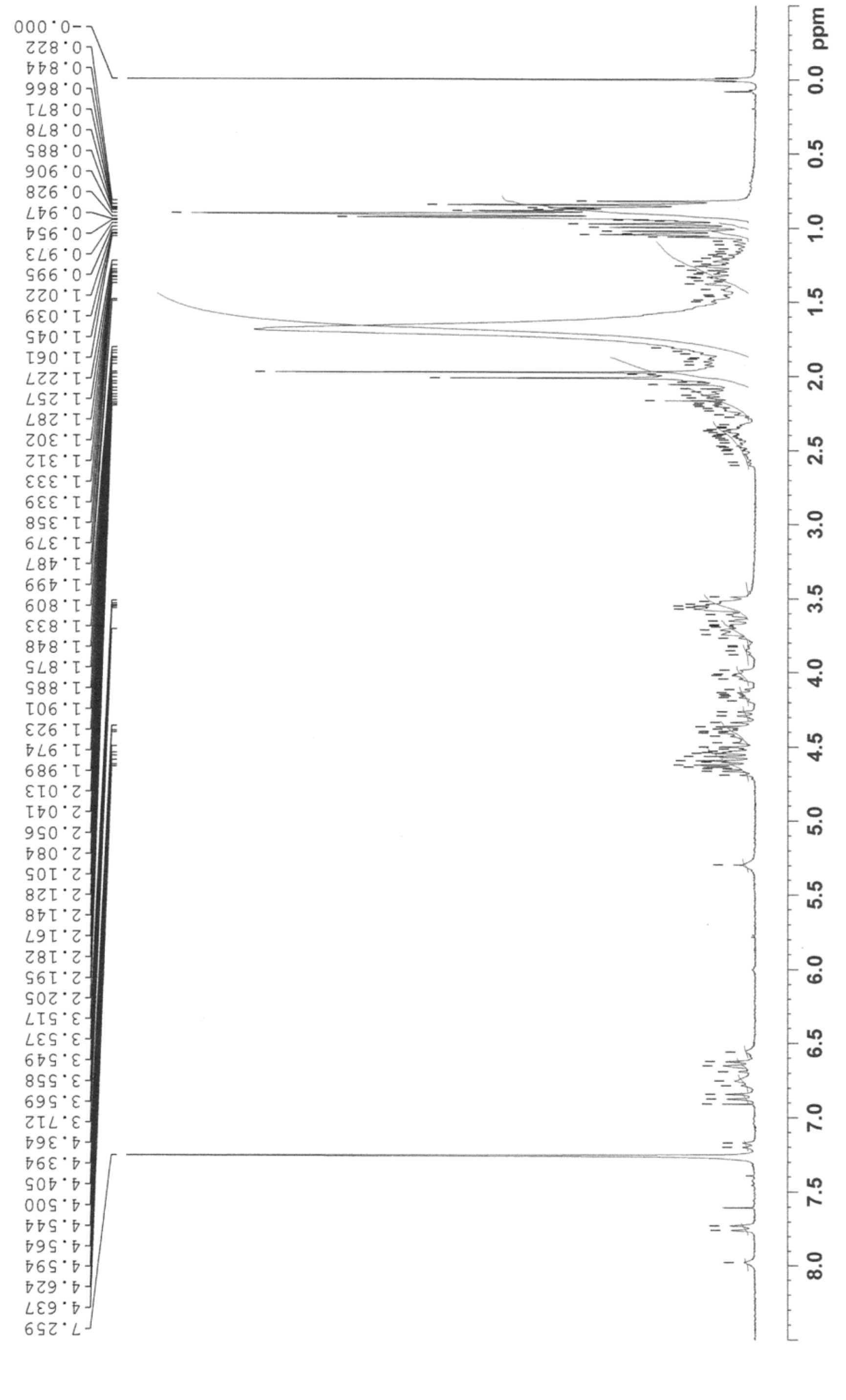

RI-peptide 3

Data File: E:¥薬品化学¥H31年度¥od smp 1-HR2.lcd

Elmt	Val.	Min	Max	Elmt	Val.	Min	Max	Elmt	Val.	Min	Max	Elmt	Val.	Min	Max	Use Adduct
H	1	65	75	O	2	7	9	Cl	1	0	0	I	3	0	0	Na
B	3	0	0	F	1	0	0	Zn	2	0	0					
C	4	35	45	Si	4	0	0	Br	1	0	0					
N	3	6	8	S	2	0	0	Sn	2	0	0					

Error Margin (mDa):	3.0	DBE Range:	not fixed	Electron Ions:	both
HC Ratio:	unlimited	Apply N Rule:	yes	Use MSn Info:	no
Max Isotopes:	all	Isotope RI (%):	1.00	Isotope Res:	10000
MSn Iso RI (%):	75.00	MSn Logic Mode:	AND	Max Results:	50

ｲﾍﾞﾝﾄ#: 1 MS(E+)　保持時間: 0.270　ｽｷｬﾝ番号: 163

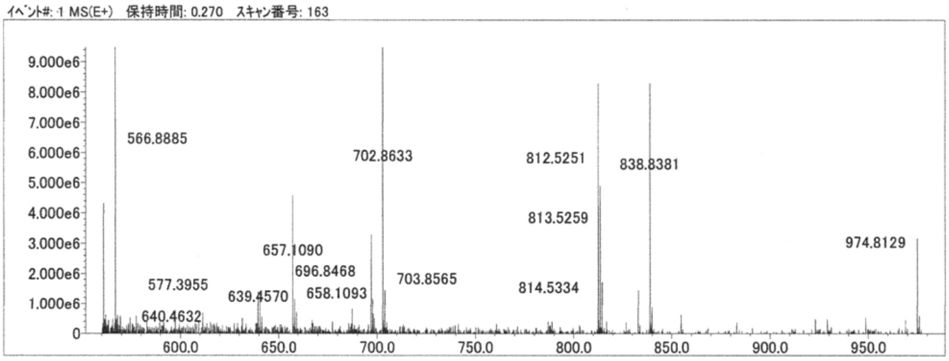

Measured region for 812.5251 m/z

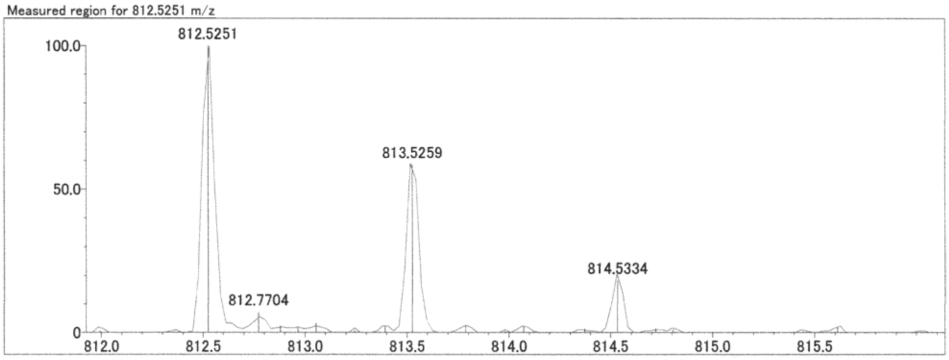

C41 H71 N7 O8 [M+Na]+ : Predicted region for 812.5256 m/z

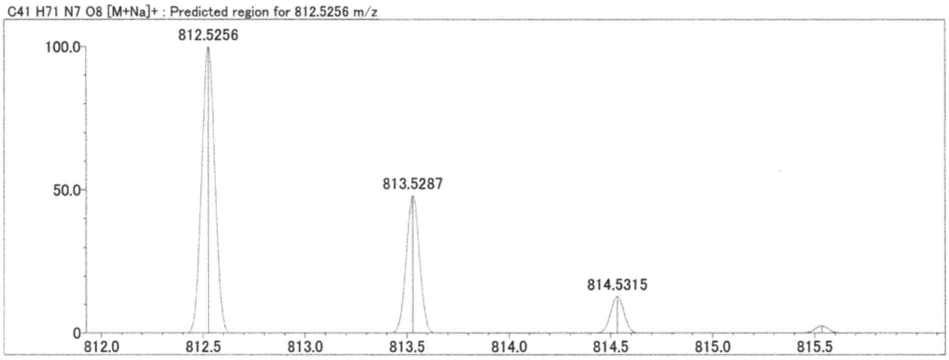

Rank	Score	Formula (M)	Ion	Meas. m/z	Pred. m/z	Df. (mDa)	Df. (ppm)	Iso	DBE
1	77.09	C41 H71 N7 O8	[M+Na]+	812.5251	812.5256	−0.5	−0.62	77.09	10.0

RI-peptide 3

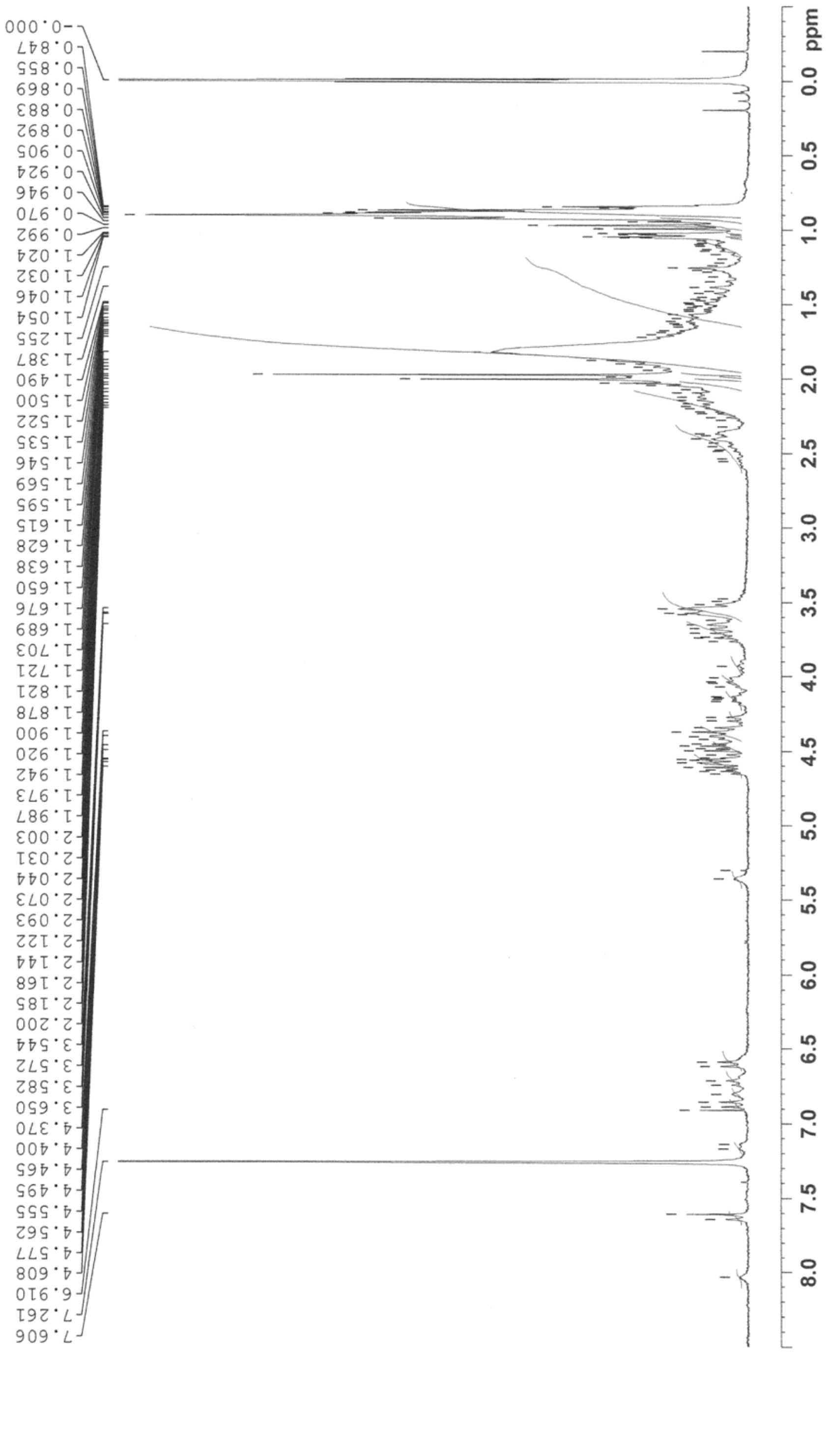

-0.000
0.847
0.855
0.869
0.883
0.892
0.905
0.924
0.946
0.970
0.992
1.024
1.032
1.046
1.054
1.255
1.387
1.490
1.500
1.522
1.535
1.546
1.569
1.595
1.615
1.628
1.638
1.650
1.676
1.689
1.703
1.721
1.821
1.878
1.900
1.920
1.942
1.973
1.987
2.003
2.031
2.044
2.073
2.093
2.122
2.144
2.168
2.185
2.200
3.544
3.572
3.582
3.650
4.370
4.400
4.465
4.495
4.555
4.562
4.577
4.608
6.910
7.261
7.606
ppm
0.0
0.5
1.0
1.5
2.0
2.5
3.0
3.5
4.0
4.5
5.0
5.5
6.0
6.5
7.0
7.5
8.0

RI-peptide 8

Data File: E:¥薬品化学¥H31年度¥od smp 2-HR3.lcd

Elmt	Val.	Min	Max	Elmt	Val.	Min	Max	Elmt	Val.	Min	Max	Elmt	Val.	Min	Max	Use Adduct
H	1	65	75	O	2	7	9	Cl	1	0	0	I	3	0	0	Na
B	3	0	0	F	1	0	0	Zn	2	0	0	Pt	2	0	0	
C	4	35	45	Si	4	0	0	Br	1	0	0					
N	3	6	8	S	2	0	0	Sn	2	0	0					

Error Margin (mDa): 3.0
HC Ratio: unlimited
Max Isotopes: all
MSn Iso RI (%): 75.00

DBE Range: not fixed
Apply N Rule: no
Isotope RI (%): 1.00
MSn Logic Mode: AND

Electron Ions: both
Use MSn Info: no
Isotope Res: 10000
Max Results: 50

ｲﾍﾞﾝﾄ#: 1 MS(E+) 保持時間: 0.397 ｽｷｬﾝ番号: 239

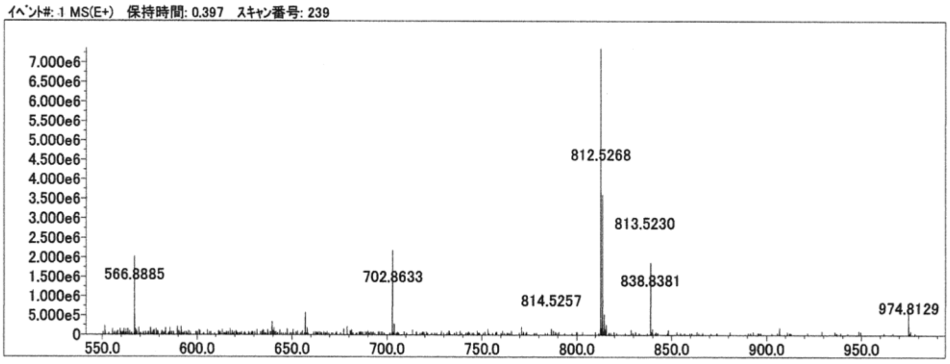

Measured region for 812.5268 m/z

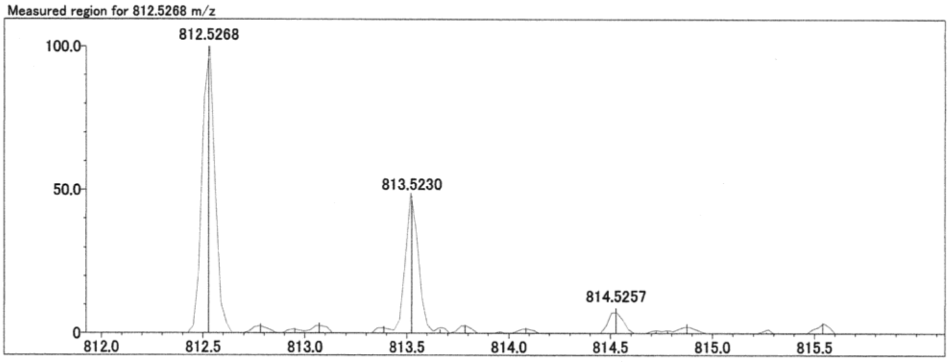

C41 H71 N7 O8 [M+Na]+ : Predicted region for 812.5256 m/z

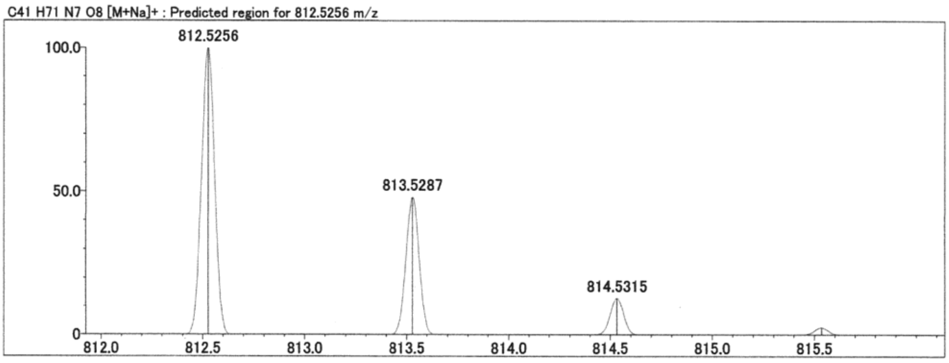

Rank	Score	Formula (M)	Ion	Meas. m/z	Pred. m/z	Df. (mDa)	Df. (ppm)	Iso	DBE
1	73.98	C41 H71 N7 O8	[M+Na]+	812.5268	812.5256	1.2	1.48	74.87	10.0

RI-peptide 8

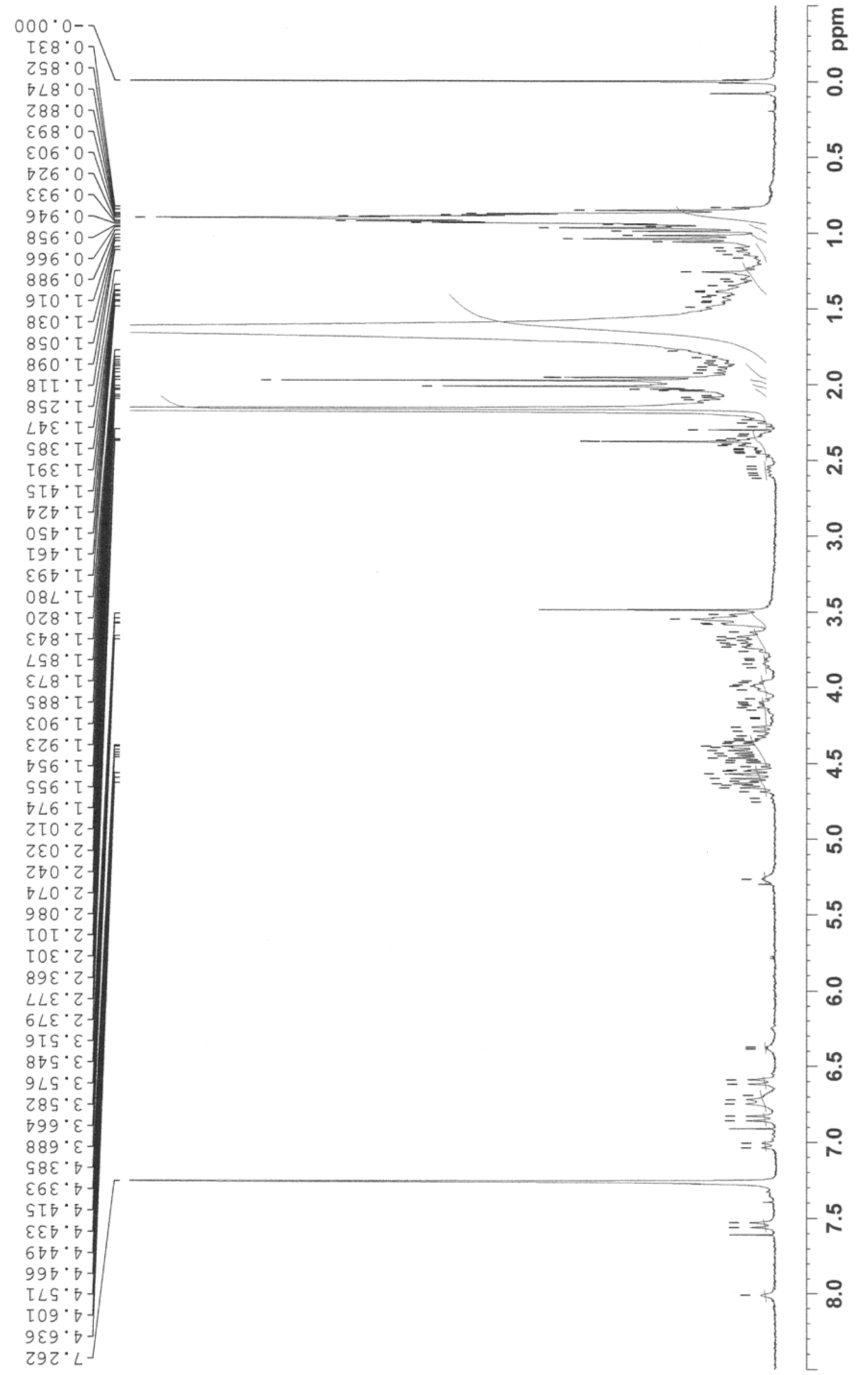

0.000
0.831
0.852
0.874
0.882
0.893
0.903
0.924
0.933
0.946
0.958
0.966
0.988
1.016
1.038
1.058
1.098
1.118
1.258
1.347
1.385
1.391
1.415
1.424
1.450
1.461
1.493
1.780
1.820
1.843
1.857
1.873
1.885
1.903
1.923
1.954
1.955
1.974
2.012
2.032
2.042
2.074
2.086
2.101
2.301
2.368
2.377
2.379
3.516
3.548
3.576
3.582
3.664
3.688
4.385
4.393
4.415
4.433
4.449
4.466
4.571
4.601
4.636
7.262
ppm
0.0
0.5
1.0
1.5
2.0
2.5
3.0
3.5
4.0
4.5
5.0
5.5
6.0
6.5
7.0
7.5
8.0

RI-peptide 9

Data File: E:¥薬品化学¥H31年度¥od smp 3-HR2.lcd

Elmt	Val.	Min	Max	Elmt	Val.	Min	Max	Elmt	Val.	Min	Max	Elmt	Val.	Min	Max	Use Adduct
H	1	65	75	O	2	7	9	Cl	1	0	0	I	3	0	0	Na
B	3	0	0	F	1	0	0	Zn	2	0	0	Pt	2	0	0	
C	4	35	45	Si	4	0	0	Br	1	0	0					
N	3	6	8	S	2	0	0	Sn	2	0	0					

Error Margin (mDa): 3.0
HC Ratio: unlimited
Max Isotopes: all
MSn Iso RI (%): 75.00

DBE Range: not fixed
Apply N Rule: no
Isotope RI (%): 1.00
MSn Logic Mode: AND

Electron Ions: both
Use MSn Info: no
Isotope Res: 10000
Max Results: 50

ｲﾍﾞﾝﾄ#: 1 MS(E+) 保持時間: 0.305 ｽｷｬﾝ番号: 184

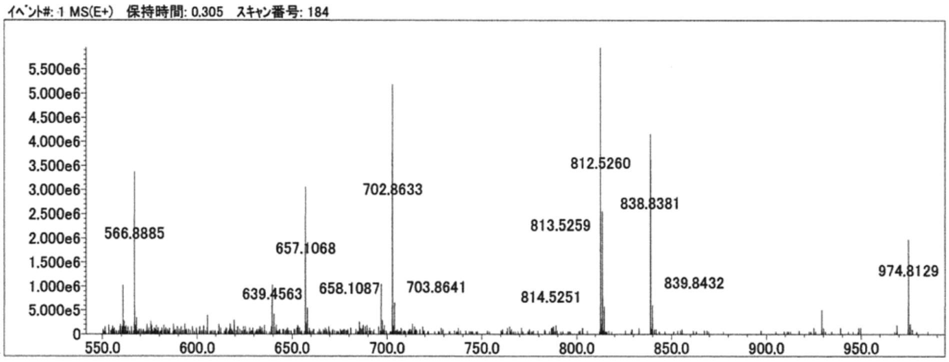

Measured region for 812.5260 m/z

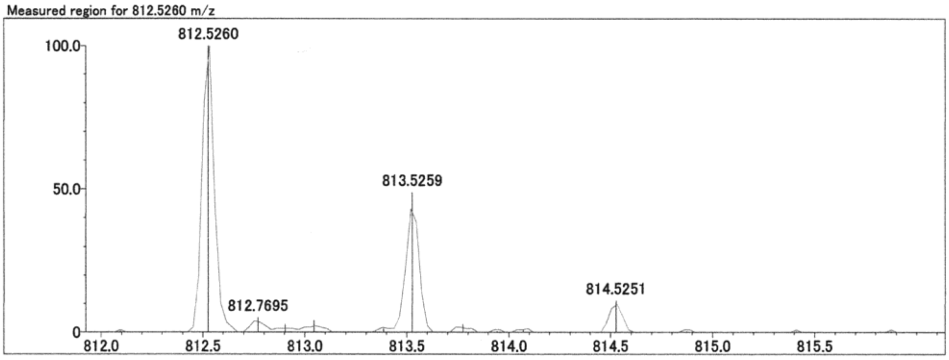

C41 H71 N7 O8 [M+Na]+ : Predicted region for 812.5256 m/z

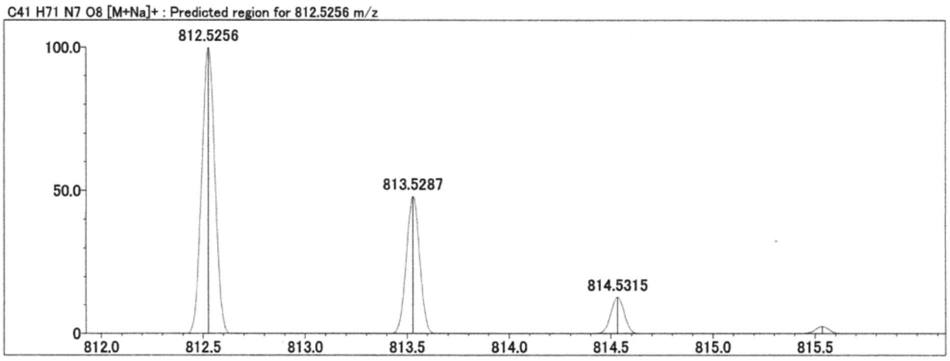

Rank	Score	Formula (M)	Ion	Meas. m/z	Pred. m/z	Df. (mDa)	Df. (ppm)	Iso	DBE
1	71.31	C41 H71 N7 O8	[M+Na]+	812.5260	812.5256	0.4	0.49	71.31	10.0

RI-peptide 9

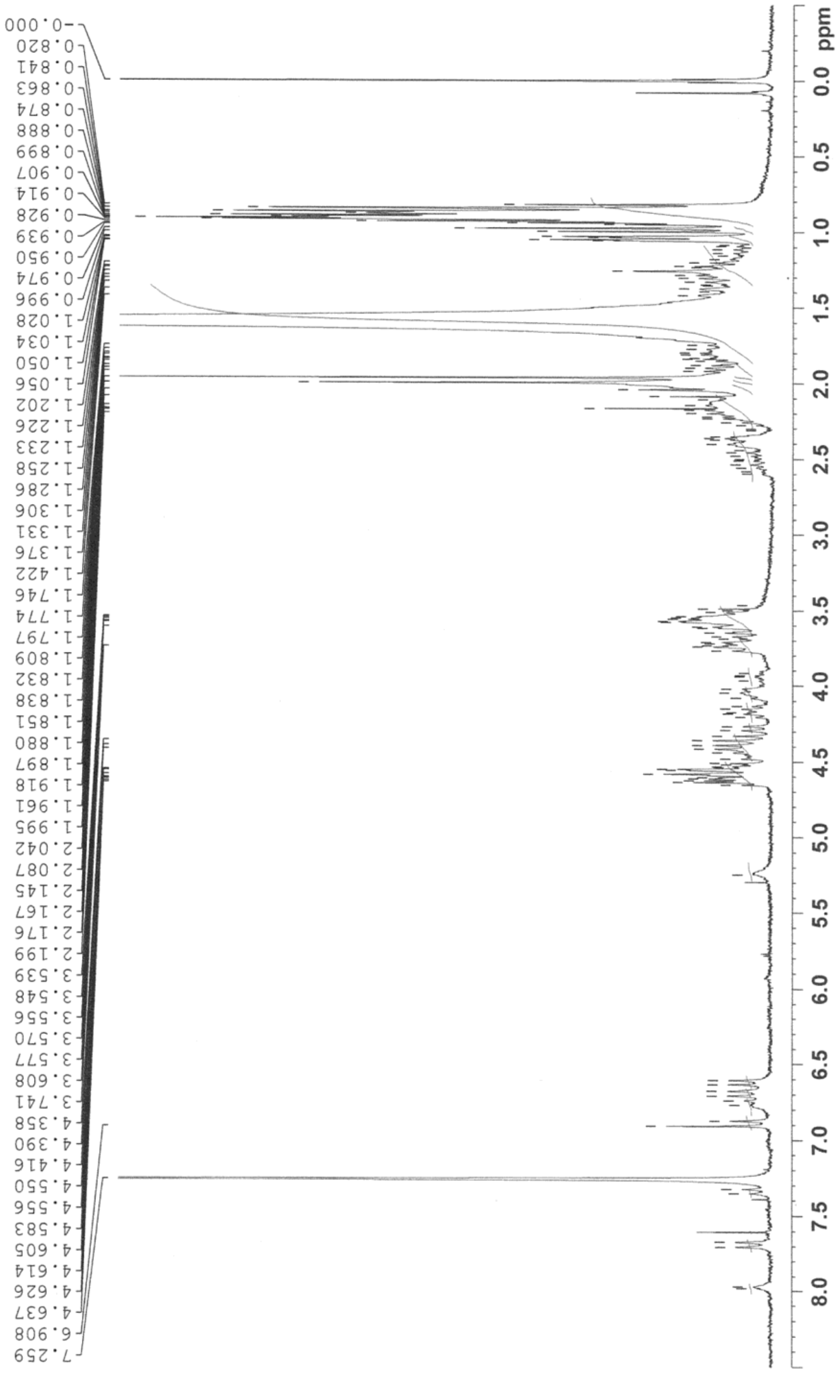
-0.000
0.820
0.841
0.863
0.874
0.888
0.899
0.907
0.914
0.928
0.939
0.950
0.974
0.996
1.028
1.034
1.050
1.056
1.202
1.226
1.233
1.258
1.286
1.306
1.331
1.376
1.422
1.746
1.774
1.797
1.809
1.832
1.838
1.851
1.880
1.897
1.918
1.961
1.995
2.042
2.087
2.145
2.167
2.176
2.199
3.539
3.548
3.556
3.570
3.577
3.608
3.741
4.358
4.390
4.416
4.550
4.556
4.583
4.605
4.614
4.626
4.637
6.908
7.259
ppm
0.0
0.5
1.0
1.5
2.0
2.5
3.0
3.5
4.0
4.5
5.0
5.5
6.0
6.5
7.0
7.5
8.0

Figure S6. Sigmoidal curve for each RI-type inhibitors

3 (D-Ile-D-Ile); IC_{50} = 240 μM

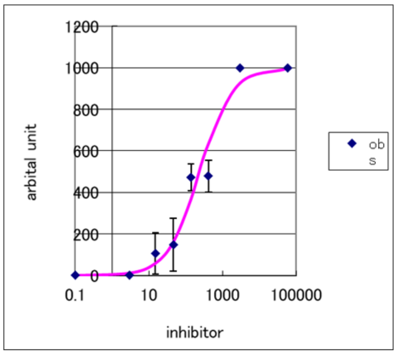

8 (D-allo-Ile-D-Ile); IC_{50} = 110 μM

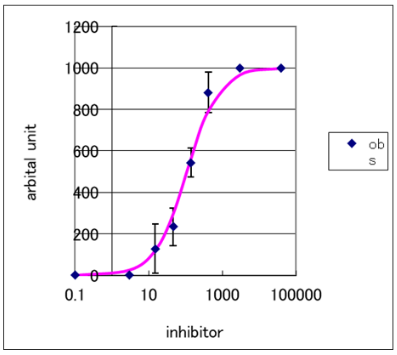

9 (D-Ile-D-allo-Ile); IC_{50} = 130 μM

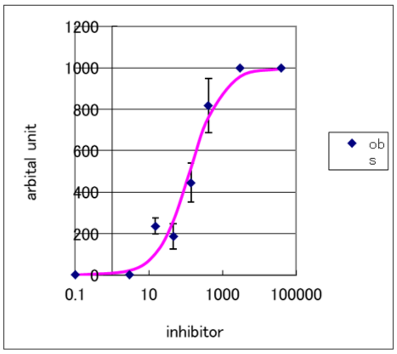

10 (D-allo-Ile-D-allo-Ile); IC_{50} = 85 μM

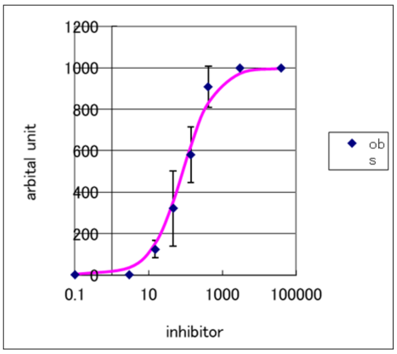

Supplement: Supplementary file 1 [file molecules-27-01646-s001.zip › molecules-1595354-supplementary.pdf]
